# Rett syndrome: a wide clinical and autonomic picture

**DOI:** 10.1186/s13023-016-0499-7

**Published:** 2016-09-29

**Authors:** G. Pini, S. Bigoni, L. Congiu, A. M. Romanelli, M. F. Scusa, P. Di Marco, A. Benincasa, P. Morescalchi, A. Ferlini, F. Bianchi, D. Tropea, M. Zappella

**Affiliations:** 1 Tuscany Rett Center, Versilia Hospital, USL Toscana Nord Ovest, Pisa, Italy; 2 Medical Genetics UOL, Ferrara University Hospital, Ferrara, Italy; 3 CNR, Istituto di Fisiologia Clinica, Pisa, Italy; 4 Neuropsychiatric Genetics, Trinity Center for Health Science, St James Hospital, D8 Dublin, Ireland; 5 Versilia Hospital, via Aurelia 335, Camaiore, Tuscany Italy

**Keywords:** RTT: Rett syndrome, MECP2: Methyl CpG binding protein 2, CDKL5: Cyclin dependent kinase-like 5, FOXG1: Forkhead box G1, Z-RTT: Preserved speech variant or “Zappella variant”, ARTT-NOS: Atypical RTT-not otherwise specified

## Abstract

**Background:**

Rett Syndrome is a neurodevelopmental disorder almost exclusively affecting females, characterized by a broad clinical spectrum of signs and symptoms and a peculiar course. The disease affects different body systems: nervous, muscolo-skeletal, gastro-enteric. Moreover, part of the symptoms are related to the involvement of the autonomic nervous system.

In the Tuscany Rett Center at Versilia Hospital, we collected data from 151 subjects with a clinical diagnosis of classical or variant RTT syndrome. For each subject, we assessed the severity of the condition with clinical-rating scales (ISS, PBZ), we quantified the performance of the autonomic nervous system, and we performed genetic analysis. We used multivariate statistical analysis of the data to evaluate the relation between the different clinical RTT forms, the cardiorespiratory phenotype, the different genetic mutations and the severity of the clinical picture. Individuals were classified according to existing forms: Classical RTT and three atypical RTT: Z-RTT, Hanefeld, Congenital. A correlation between C-Terminal deletions and lower severity of the clinical manifestations was evident, in the previous literature, but, considering the analysis of autonomic behaviour, the original classification can be enriched with a more accurate subdivision of Rett subgroups, which may be useful for early diagnosis.

**Results:**

Present data emphasize some differences, not entirely described in the literature, among RTT variants. In our cohort the Z-RTT variant cases show clinical features (communication, growth, epilepsy and development), well documented by specific ISS items, less severe, if compared to classical RTT and show autonomic disorders, previously not reported in the literature. In this form epilepsy is rarely present. In contrast, Hanefeld variant shows the constant presence of epilepsy which has an earlier onset In Hanefeld variant the frequency of apneas was rare and, among the cardiorespiratory phenotypes, the feeble type is lacking.

**Conclusion:**

A quantitative analysis of the different autonomic components reveals differences across typical and atypical forms of RTT that leads to a more accurate classification of the groups. In our cohort of RTT individuals, the inclusion of autonomic parameter in the classification leads to an improved diagnosis at earlier stages of development.

## Background

Rett Syndrome (RTT) is a neurodevelopmental disorder almost exclusively affecting females, characterized by a broad clinical spectrum of signs and symptoms and a peculiar course during life. It occurs with an average frequency of about 1:10,000 in girls [1].

After the first identification of the gene in 1999 [2], *MECP2* mutations have been identified in 90–95 % of the Classical RTT cases, where, after a period of apparently normal neuropsychic development with some alterations in the general movements [3], individuals after 6–18 months present an arrest in their development followed by a progressive deterioration of acquired skills such as purposeful hand function and communication, a deceleration of head growth, with the subsequent appearance of stereotypic hand movements. This syndrome is associated with comorbidities including reduced somatic growth, gastro-intestinal problems, osteopenia, gait apraxia, scoliosis, autonomic dysfunction, breathing disturbances and frequent seizures.

According to the new revised criteria, it has been recognized that some individuals present many of the clinical features of RTT, such as regression, but do not fit the criteria established for the diagnosis of Classical RTT [4]. These have been termed “atypical” RTT [5].

Among RTT atypical forms, some specific variants have been described: Preserved Speech Variant or “Zappella variant” (Z-RTT), characterized by milder clinical abnormalities and by the appearance of some degree of speech [6, 7]; the “Hanefeld variant” (CDKL5 gene related) in which there is a pathognomonic early onset of seizures [8, 9]; the “Congenital variant” (FOXG1 gene related), with onset of the symptoms since after birth [10]. In these last two variants, the mutation and several clinical features are notably different, hence they are considered as distinct clinical and molecular entities [11].

All these forms have different severity degrees in terms of comorbidities, behaviour, prognosis and involvement of the autonomic nervous system. The present study describes the clinical and autonomic features of the RTT girls who attend the Tuscany Rett Center, in order to obtain more information on the correlation between genotype and phenotype.

## Methods

Our cohort consists of 151 individuals evaluated in the Tuscany Rett Center, Versilia Hospital (Lido di Camaiore, Italy) from January 2006 to April 2014. During the study six girls died due to complication of illnesses or sudden death or because of drug resistant infections.

Diagnosis of RTT (Classical, Atypical) was made by two independent child neuropsychiatrists with expertise on RTT, and individuals were categorized on the basis of the RTT consensus revised criteria [4] 64.9 % of the girls presented classic Rett, while 35.1 % presented atypical RTT. 20 % of the individuals presented evident autistic signs.

In our cohort all the forms described in the current classification [4] were present, considering the wide variability of the RTT phenotype, the age of onset, the severity of impairments, and the clinical course. We also observed some different type of “Rettoid” variants that are now described as RTT spectrum, and were previously classified as “forme fruste”: late childhood regression variant, Rettoid Male, familial atypical variant [12].

In our cohort there are also individuals that can be classified as atypical RTT, but they do not belong to any of the specific forms. These individuals are two boys and four girls with MECP2 mutations, one female with MEF2C mutation and two girls with no mutation. These individuals have been grouped in variant cases and they will be referred to as Atypical RTT not otherwise specified (ARTT-NOS).

Phenotypic data were collected through clinical and neurological evaluations. In all RTT cases, cardiorespiratory parameters and brain activity were investigated. For each patient we assessed: clinical severity, cardiorespiratory parameters, brain activity, and genetic mutation.

### Clinical assessment

For clinical assessment of the severity degree, all individuals were evaluated using both the International Scoring System (ISS) [13], and the Pini Bonuccelli Zappella scale: PBZ [14].

International Scoring System (ISS): The ISS scale consists of 21 items regarding the RTT typical features, divided in five subscales: Growth and Development, Musculoskeletal, Movement, Mental-Cortical and Brainstem-Autonomic. Each item score ranges from 2 to 0 as follows: 2 severe abnormality, 1: mild abnormality, 0: no abnormality. Lower the score, the better the clinical condition. The total score range between 0 and 15 has been considered as mild, between 16 and 24 as moderate and above 24 as severe.

Pini Bonuccelli Zappella (PBZ): The PBZ scale takes into account specific items about daily skills, and consists in 26 items grouped into 3 functional domains: movement (MOV): facial expressions, eye contact, praxis, stereotipies, walking, sitting position, muscular tone, tropism and dystonias and other movement disorders; communication and sensitive (C-S) speech production, comprehension, attention to visual, tactile, and auditory stimuli, breathing, convulsions, appetite, sleep, night-time sleep, day-time sleep, aggressivity; daily living (DL) climbing and, descending stairs, feeding/swallowing, feeding autonomy, falls unrelated to seizures. Each item score ranges from 0 (normal) to 4 (more severe). The total score ranges between 0 and 23 (mild), 24–37 (moderate), 38–49 (severe) and > 49 (very severe). For the abstract follow the link: http://rivistedigitali.erickson.it/autismo/visualizza/articolo/11690/index.html.

#### Cardio-respiratory assessment

All the individuals have been investigated with the Neuroscope system (Medifit Instruments Ltd, London, UK) [15] to analyse their autonomic functions.

The NeuroScope™ employs VaguSoft software (MediFit Diagnostics Ltd, London, UK), allowing to record simultaneously several autonomic cardiovascular and respiratory parameters non-invasively and synchronously with EEG and video monitoring over a 1-h period. The autonomic parameters included: cardiac vagal tone, heart rate, transcutaneous blood gases (TCM4, Radiometer, Copenhagen, Denmark), and respiratory patterns. Thirteen abnormal, awake breathing rhythms have been identified in the RTT syndrome: Breath hold, Central apnoea, Rapid and shallow breathing, Hyperventilation, Tachypnoea, Deep breathing, Biot’s breathing, Cheyne-Stokes breathing, Regular breath holds, Protracted inspiration, Atypical breathing, Shallow breathing and Valsalva’s manoeuvre. Following analysis of the recording each patient was assigned to a specific cardiorespiratory phenotype according to the predominant pattern of respiratory dysrhythmia [16].

Three abnormal breathing rhythms in the waking state have been identified in the RTT population and categorised into Feeble, Forceful, and Apneustic type of breathings. They constitute three unique cardiorespiratory phenotypes with different levels of blood gases, autonomic tone, physical features, clinical complications and idiosyncratic responses to drugs [17].

The cardio-respiratory assessment has been performed in the Tuscany Rett Center only on individuals with confirmed clinical and genetic diagnosis.

### Genetic analysis and mutations classification

Genetic analysis has been performed on all the individuals with clinical RTT phenotype clinical presentation, first for MECP2, (sequencing of the coding region and Multiple Ligand Probe Amplification) and, whenever negative or in case of early infantile onset epilepsy for CDKL5. In two females FOXG1 and MEF2C molecular analysis was performed upon specific clinical suspicion [18, 19]. The majority of genetic tests have been performed in different molecular laboratories. All MECP2 and CDKL5 mutations have been classified into 3 principal categories according to the nature of the sequence variation and its subsequent effect on the protein sequence: substitutions (nonsense, missense), deletions and frameshift. Among the frameshift mutations we focused on the mutations located in the C-terminal segment of the gene. The group of the nonsense mutations is furthermore divided into early truncating and late truncating mutations according to the position of the mutation in the gene [20].

### Description of the sample and of the different clinical RTT forms

Our sample consists of 151 individuals: 149 females and 2 males, 118 individuals present mutations in MECP2, 12 females are mutated in CDKL5, 1 in FOXG1 and 1 in MEF2C. In 19 individuals all the molecular analysis resulted negative. The mean age in our cohort is 12 years (range 1–49, median 10).

Genetic tests and instrumental evaluations have been performed in all individuals. However, for some few individuals, some data (like auxological parameters at birth or development in the first year of life) were lacking. This is the reason why the tables below have different numbers.

The different clinical RTT forms in our sample are: 98 classical RTT (64.9 %) and 33 Atypical RTT (35.1 %). Among the Atypical RTT cases, 19 females (12.6 %) present with Z-RTT variant, 13 (8.6 %) with Hanefeld variant, 1 (0.7 %) with congenital variant, and 20 cases with ARTT-NOS variant (13.3 %). For each group we report %, mean age at clinical evaluation, and mean age of diagnosis (Table 1).

**Table 1:** Sample description—Clinical forms, age at the evaluation and age at diagnosis

Clinical forms	N.	%	Age at the evaluation Mean (SD)	Age at the diagnosis Mean (SD)
Classical	98	64,9	12,5 (10,2)	6,7 (8,5)
Z-RTT	19	12,6	12,5 (7,9)	7,3 (5,6)
Hanefeld	13	8,6	6,1 (4,8)	2,6 (1,7)
Congenital	1	0,7	2	1
ARTT-NOS	20	13,2	13,5 (9,5)	8,45 (4,8)
Total	151	100	–	–

### Statistical analysis

Data are described as mean and standard deviation (SD), median and percentiles for continuous variables and as absolute and relative frequencies for categorical variables.

Parametric analysis (ANOVA with Bonferroni) for continuous variables and the *χ*^2^ test or Fisher’s exact test for categorical variables, were used to measure differences between groups.

*P* value < or equal to 0.05 were considered statistically significant and all *P* values were based on two tailed tests. Statistical analysis was performed using STATA 9.0 for Windows (StataCorp, College Station, TX).

## Results

### Evaluation with the International Scoring System (ISS)

The total ISS score has been calculated for 144/151 (95 %) RTT cases, since for 7/151 RTT cases the clinical information was not complete (i.e. early growth and developmental data lacking). The mean total ISS score was 18.8. For classical forms (*n* = 98) ISS = 19.5 ± 5.9; Z-RTT (*n* = 19) ISS = 11.7 ± 3.7; Hanefeld (*n* = 11) ISS = 19.4 ± 4.8; Congenital (*n* = 1) ISS = 26; ARTT-NOS (*n* = 19) ISS = 21.4 ± 6. These differences are statistically significant (F test = 9.33; *p* < 0.001) (Table 2) and confirm that the Z-RTT females have the mildest presentation if compared to all the other RTT forms [6].

**Table 2:** Clinical forms and ISS total score, percentage

ISS total	% Classical (*n* = 98)	% Z-RTT (*n* = 19)	% Hanefeld (*n* = 13)	% Congenital (*n* = 1)	% ARTT-NOS (*n* = 20)
Mean (SD)	19.5 (5.9)	11.7 (3.7)	19.4 (4.8)	26	21.4 (6)

### ISS (subscales and items) and clinical forms

In order to evaluate the clinical severity of the common features in the RTT disorder, a modified version of the International Scoring System was used [16]. The ISS scoring list consists of 21 features divided over five functional domains: Growth and Development domain (DEV); Musculoskeletal domain(M_SKEL); Movement domain (MOV); Cortical domain(CORT), Autonomic domain (BRAIN).

The oro-motor disturbances, initially considered part of movement domain, were included in the Autonomic domain, according to Julu and Witt Engerström [16]. The following tables show the ISS scores according to the different functional domains in the different clinical forms.

#### ISS subscales with statistical significance and clinical forms

- Growth and development domain
  All the auxological parameters: weight, height, head circumference (OFC) and Body-Mass Index (BMI) of all RTT cases were significantly reduced if compared to the normal population but they were higher in the Z-RTT variant (F test = 8.18; *p* < 0.005; Table 3) than in the classical form and Hanefeld variant (except for OFC). In fact in only the 5–10 % of the Z-RTT variant cases, height, weight, OFC were below the 3rd centile [21]. The ARTT-NOS group presents, on the contrary, a major impairment in growth if compared to the other forms.

**Table 3:** Subscale growth and development and clinical forms

	% Classical (*n* = 97)	% Z-RTT (*n* = 19)	% Hanefeld (*n* = 12)	% ARTT-NOS (*n* = 20)
Height				
<3rd centile	39	5	17	60
3^rd^–40^th^ centile	48	47	58	15
>40^th^ centile	13	48	25	25
Weight				
<3^rd^ centile	40	10	17	60
3^rd^–40^th^ centile	35	21	58	20
>40^th^ centile	25	69	25	20
OFC				
<3^rd^ centile	46	5	8	55
3^rd^–40^th^ centile	39	27	50	30
>40^th^ centile	15	68	42	15
BMI				
<3^rd^ centile	43	1	27	53
3^rd^–40^th^ centile	23	16	45	16
>40^th^ centile	34	79	27	31
  We found a significant association between Rett clinical forms and BMI (*χ*^2^ test = 19.54; *p* < 0.05). In detail, the Z-RTT has a different centile distribution if compared to other forms: classical, Hanefeld and ARTT-NOS forms have a BMI lower than the 40^th^ centile in the majority of cases while the Z-RTT have, in a large majority of cases (about 80 %) a BMI above the 40^th^ centile.
  The BMI value was significantly higher in the Z-RTT variant cases compared to the Classical and the ARTT-NOS form (F test = 4.77; *p* < 0.005).
- Musculoskeletal domain
  This investigation considered three parameters: muscular tone, contractions, and scoliosis. We reported the data for the spinal curvature since is the only parameters with significant differences across clinical forms.
  Scoliosis
  In our sample, 85/151 subjects presented scoliosis. Among these, the degree of scoliosis is severe in 31 (36 %) and mild in 54 (63 %); *χ*^2^ = 15.4, *P* = 0.052, Table 4.

**Table 4:** Scoliosis and clinical forms

Scoliosis	% Classical (*n* = 98)	% Z-RTT (*n* = 19)	% Hanefeld (*n* = 13)	% Congenital (*n* = 1)	% ARTT-NOS (*n* = 20)
No deviation	36.7	63.1	75	100	35
Mild	36.7	31.6	23.1	–	45
Severe	26.5	5.26	–	–	20
  Scoliosis was more present in the RTT classical sample if compared to the Z-RTT and to the Hanefeld (*χ*^2^ test = 15.4; *p* = 0.05). In the subgroup of the RTT not walking (28.5 %), the scoliosis was severe in the 50 % of the sample.
- Movement domain
  We analysed four main areas of motor function: walking, use of hands, stereotypes and other movement disorders (Table 5).

**Table 5:** Movement domain and clinical forms

Walking	% Classical (*n* = 97)	% Z-RTT (*n* = 19)	% Hanefeld (*n* = 13)	% Congenital (*n* = 1)	% ARTT-NOS (*n* = 20)
Walks	21.6	36.8	15.4	–	10
Walking impaired	50.5	57.9	38.5	–	55
Not walking	27.8	5.3	46.1	100	35
Use of hands	% Classical (*n* = 97)	% Z-RTT (*n* = 19)	% Hanefeld (*n* = 13)	% Congenital (*n* = 1)	% ARTT-NOS (*n* = 20)
Normal	2	15.8	–	–	–
Reduced	54.6	78.9	61.5	–	65
No	43.4	5.3	38.5	100	35
Stereotypies	% Classical (*n* = 95)	% Z-RTT (*n* = 19)	% Hanefeld (*n* = 13)	% Congenital (*n* = 1)	% ARTT-NOS (*n* = 20)
Absent	4.2	21.05	15.4	–	15
Mild	46.3	42.1	61.5	100	50
Dominant or constant	49.5	36.8	23.1	–	35

  Walking ability
    Since the walking can start either before or after regression stage, and it can be lost in stage IV of the disease, we reported the status at the stage of evaluation.
    Among the 151 subjects, 32 (21.2 %) were able to walk without support, 76 (50.3 %) with support and 43 (28.5 %) were not able to walk. We found that females with Z-RTT variant were more able to walk without support if compared to females with classical and Hanefeld forms. Statistical analysis revealed that only 5 % of Z-RTT girls could not walk, while 45 % of Hanefeld cases could not walk (*χ*^2^ test = 7.75; *p* = 0.021).
    In the subgroup of the RTT not walking, the scoliosis was severe in the 50 % of the sample.
  Use of hands
    The evaluation of use of hands ability revealed that only the 3.3 % of the individuals were able to adequately manipulate an object; 59.3 % could grab and hold the object just for few seconds; 37.3 % of the cases did not have any purposeful use of the hands.
    The dyspraxia was more severe in the group of the classical RTT (43.4 %), ART-NOS (35 %) and Hanefeld variant (38.5 %) compared to Z-RTT (5.3 %), (*χ*^2^ test, *p* = 0.01), in Table 5. Even considering the average across all the different groups (42 %) Z-RTT females showed lower impairment (5.3 %). Dispraxia was significantly different in females with Z-RTT (5.3 %) versus classic RTT (43 %) (*χ*2 test = 14.75, *p* = 0.001).
  Stereotypies
    The presence of stereotypies is particularly important in the diagnosis of RTT syndrome. Initially stereotypies are limited to bringing the hand to the mouth, and later appear typical bimanual stereotypies different from those observed in autism.
    Hand stereotypies were present in 135/148 RTT subjects and had a higher occurrence in the RTT classical forms than in all the variant forms: in particular stereotypies were absent in only 4 % of Classic RTT individuals, while 21 % of Z-RTT females failed to show stereotypies (*χ*^2^ test = 6.87, *p* = 0.032).
  Other movement disorders (tremors, dystonia and similar)
    The 50.3 % of the RTT cases showed some movement disorders, often tremor. In the 23.2 % this tremor was mild, in the 0.7 % instead it was sub-continuous and disabling. No significant statistically differences were observed among the groups.
- Cortical domain
  Cortical investigation is related to three main parameters: intellectual disabilities, language impairment and epilepsy. We analyzed intellectual impairment, language and epilepsy. For epilepsy, we considered its onset and its distribution across clinical forms. For cognitive level and language, we could only use two scores: profound or mild impairment, since there were no girls with a “no compromise” value. However there were some Z-RTT girls able express themselves relatively well, with extensive vocabulary and spontaneous construction of sentences.
  Speech
    Since the first description of Andreas Rett one of the key symptoms was the absence or loss of speech. At the onset of the disease some children had already developed a vocabulary of a few words or even sentences with two words. To this stage usually follows a stop phase and then regression, however 12.6 % of females in our sample recovered and amplified the vocabulary, using more than 10 words at age of 5; they constitute the Zappella variant. In this form we recognized the presence of moderate speech consisting of a few words, and eventually the construction of complex sentences, sometimes echolalic, sometimes purposeful, with an extensive vocabulary. In some the language skills extended to the understanding of writing.
  Intellectual impairment
    The disorder involves a significant cognitive impairment, very difficult to quantify in standard tests: the lack of speech and the eminent dyspraxia implies that many assessments should be done with eye tracking technology or other techniques of augmentative and alternative communication (AAC). These methods are incompatible with short assessments, carried out in our sample. ISS discriminates between profound cognitive impairment (IQ <20) and cognitive impairment present but not profound (Q.I. between 20 and 70), because by definition there are no cases with normal cognitive levels.
    The distinction that we have arbitrarily used is the acquisition of object consistency and attention to language and images. According to this criteria we distinguished a profound cognitive delay for individuals with IQ <20, and a mild cognitive delay in individuals with IQ between 20 and 70.
    Z-RTT females showed minor globally impairment that others groups (F test = 21.12; *p* < 0.001).
  Epilepsy
    We analysed epilepsy distribution and onset across the clinical forms.
    In the whole sample, epilepsy was present in 64.2 % of cases. Epilepsy was present in the 58.2 % of classical RTT cases and in all the cases with the Hanefeld variant (100 %). Drug resistant (completely or partial) epilepsy was present in 17.2 % of cases.
    Furthermore, we observed that the drug resistant epilepsies had progressively higher percentages in the following order subgroups: Z-RTT variant 5.3 %; ARTT-NOS 15 % Classical RTT 17.4 % and Hanefeld variant 38.5 %. Severe epilepsy was present with major frequency in Hanefeld variant if compared to the other clinical forms (15.3 %) with *χ*^2^ test = 15.7; *p* < 0.05 (Table 6).

**Table 6:** Epilepsy and clinical forms

Epilepsy	% Classical (*n* = 98)	% Z-RTT (*n* = 19)	% Hanefeld (*n* = 13)	% Congenital (*n* = 1)	% ARTT-NOS (*n* = 20)
Absent	41.8	36.8	–	100	25
Controlled by therapy	40.8	57.9	61.5	–	60
Barely or not controlled by therapy	17.4	5.3	38.5	–	15
    The median age of onset of epilepsy in the entire sample was 50 months with a range between 1 and 216 months. An earlier onset epilepsy was present peculiarly in the Hanefeld variant compared to the other forms (F test = 9.38, *p* < 0.001) (Table 7).

**Table 7:** Epilepsy distribution and onset (in months) and clinical forms

Epilepsy onset	Classical (*n* = 51)	Z-RTT (*n* = 11)	Hanefeld (*n* = 13)	Congenital (*n* = 0)	ARTT-NOS (*n* = 13)
Mean (SD)	62.6 (43)	49.4 (11.7)	2.2 (1.6)	–	49 (39.9)
Range	1–216	36–72	1–7	–	2–108
- Autonomic domain
  The autonomic Nervous system is related to several abnormalities: impaired swallowing, peripheral circulation in limbs, mood disorders, sleep disorders, abnormalities in the gastro-esophageal tract, urinary bladder and cardio-respiratory abnormalities (see below: Respiratory abnormalities and cardiorespiratory phenotypes). Here we report gastro-enteric dysfunction and sleep (Table 8).

**Table 8:** Feeding problems and clinical forms

	% Classical (*n* = 96)	% Z-RTT (*n* = 19)	% Hanefeld (*n* = 13)	% Congenital (*n* = 1)	% ARTT-NOS (*n* = 20)
Dysphagia					
None	35.4	58	38.5	–	40
Mild	47.9	42	31	100	50
Severe	16.7	–	30.5	–	10
Constipation					
None	23.9	47.4	23.1	–	20
Mild	59.4	36.8	53.8	100	55
Severe	16.7	15.8	23.1	–	25

  Gastro-enteric disorders
    RTT syndrome is often associated with nutritional intake and growth problems. In general, feeding difficulties are complex and involve oromotor, behavioral, nutritional and medical components. Oromotor problems may include: oropharyngeal dysfunction, sensory defects, reduced mobility of the tongue. One or all phases of the deglutition function can be compromised.
    Furthermore, breath disorders could disturb the swallowing ability; drug used to control epilepsy can induce lack of appetite, sedation and increase of drooling. Severe not treated constipation can influence in a bad way the appetite too. In our cohort use of PEG was rare (2 %).
    The 38.9 % of the individuals did not present dysphagia, 46.3 % presented dysphagia for solid foods but not for liquid ones, while only the 14.7 % presented dysphagia for both. Among Z-RTT females, nobody presented severe dysfagia and in 58 % disfagia was absent. Sixty-two percent individuals with Hanefeld variant presented functional dysfunction with half individuals with severe dysfunctions and half with mild dysfunctions. There was a significant difference between groups (*χ*2 test = 6.69; <0.035).
    In our sample more than a half of the cases (55.7 %) presented a mild irregular intestinal rhythm and 18.1 % of individuals presented severe constipation; many of these individuals needed drug based approach. The Z-RTT females presented constipation more rarely than the other groups.
  Sleep
    There is high prevalence of sleep problems in Rett syndrome (80–94 % across different age groups). Sleep problems were identified most frequently in the younger age group. Daytime napping, night-time laughter, teeth grinding, night screaming and seizures were the problems most frequently reported. Some specific sleep problems did appear to vary with age with night laughing decreasing with age and daytime napping increasing with age. Sleep dysfunction had consequence on the whole family, hence in our cohort we considered only the most severe cases with sleep difficulties and early awakenings.
    Sixty-one point seven percent of the individuals did not present sleep disorders, the 24.8 % presented occasional difficulties and only the 13.4 % of the cases needed drugs to treat them. There was a significant statistical difference (*χ*^2^ test = 17.7; *p* < 0.02, Table 9) in Z-RTT females the sleep abnormalities were less severe.

**Table 9:** Sleep and clinical forms

Sleep	% Classical (*n* = 96)	% Z-RTT (*n* = 19)	% Hanefeld (*n* = 13)	% Congenital (*n* = 1)	% ARTT-NOS (*n* = 20)
None	66.7	78.9	38.5	–	40
Mild	17.7	21.1	46.1	100	45
Severe	15.6	–	15.4	–	15
    The 69 % with Classical forms and Z-RTT did not show any alteration in the sleep-awake rythm compared to other subgroups (38 %).
    We report cardio-respiratory abnormalities later, since they are often misinterpreted by caregivers.
  ISS areas and clinical forms
    The results of the ISS components in the different forms are reported below. The table was realized with the individuals with reported scores for ISS (Table 10).

**Table 10:** ISS areas and clinical forms

ISS areas		Classical	Z-RTT	Hanefeld	Congenital	ARTT-NOS
Development (*n* = 144)	Mean (SD)	4.2 (2.6)	1.4 (1.4)	4.4 (2.6)	9 (−)	5.5 (2.8)
	Range	0–8	0–5	2–8	–	0–9
M_SKEL (*n* = 146)	Mean (SD)	2.6 (1.8)	1.2 (1.1)	1.5 (0.9)	2 (−)	2.4 (1.7)
	Range	0–6	0–5	0–3	–	0–5
Movement (*n* = 147)	Mean (SD)	4.6 (1.4)	3.4 (1.3)	4.5 (1.1)	6 (−)	4.5 (1.5)
	Range	0–8	1–6	3–6	–	2–8
Cortical (*n* = 146)	Mean (SD)	3.8 (1.1)	2.5 (0.8)	5.1 (1)	4 (−)	3.9 (0.9)
	Range	1–6	1–5	3–6	–	3–6
Brain (*n* = 146)	Mean (SD)	4.3 (2)	3.3 (1.8)	4.5 (1.8)	5 (−)	5.1 (2.4)
	Range	1–9	0–7	2–8	–	1–10
    Growth and development: Z-RTT showed a minor deficit than all the other RTT form (F test = 8.18; *p* < 0.005). Musculoskeletal appearance: Z-RTT group was less affected than the Classical RTT group (F test = 3.29; *p* < 0.05). Movement: the motricity area was less compromised in the Z-RTT if compared to the classical forms (F test = 3.5; *p* < 0.005). Cortical: minor impairement was observed for the group of Z-RTT if compared to the ARTT-NOS, Hanefeld, (F test = 12.21; *p* < 0.001). Autonomic system: no significant differences among the single RTT clinical forms. Altogether we confirmed that Z-RTT females have lower scores as they have a milder clinical presentation.

### Evaluation with Pbz scoring system

#### PBZ and clinical forms

##### PBZ

The PBZ is a scale of evaluation designed to test severity in Classical Rett and its variants. PBZ scoring scale, as ISS, considers motor performances and sensitive and comunicative abilities (S-C), and in addition, takes into account daily living. This scoring system has a similar trend of ISS, but measures also daily living and it is more sensible for the association analysis with genetic mutations. The scale is susceptible to recording even minimal changes in clinical symptoms over the course of therapy, rehabilitation programmes and environmental enrichment, but also to indicating the severity of different clinical and genetic groups (Table 11).

**Table 11:** PBZ areas mean scores and clinical forms

PBZ score		Classical	Z-RTT	Hanefeld	Congenital	ARTT-NOS
Motor area	Mean (SD)	13.3 (4.6)	6.4 (3.8)	16.3 (4.5)	0 (−)	11.8 (2.3)
	Range	5–24	0–15	12–22	–	8–14
S-C	Mean (SD)	12.2 (4.6)	8 (4.1)	21.5 (5.7)	–	14.2 (5.9)
	Range	3–23	1–15	16–32	–	8–25
Daily living	Mean (SD)	10.2 8 (3.4)	5.4 (3.4)	11.8 (2.7)	–	7.7 (2.8)
	Range	1–17	0–11	7–15	–	4–13
Total	Mean (SD)	35.7 (9.8)	19.8 (10.3)	49.7 (11.2)	–	33.7 (6)
	Range	13–62	2–41	40–69	–	27–44

Females with Hanefeld variant presented the worst data for ISS and daily living.

Table 12 shows the various classes of PBZ for Classic Rett and variants: the differences were all significant in each area: Motor area: all forms were significantly more severe than the Z-Rett (*χ*^2^ test = 40.53; *p* < 0.001). Communicative and sensory skill area (SC): in a total of 6 females with Hanefeld presentation, 3 variant were severe and 3 very severe, and showed a significant difference versus all other (χ^2^ test = 25.93; *p* < 0.01), while the Z-Rett rank mainly between mild forms (50 %) and medium (36 %).

**Table 12:** PBZ and severity in clinical forms, percentage

PBZ areas	Severity scale	Classical	Z-RTT	Hanefeld	ARTT-NOS
Motor (0–36)	Mild	3.8	50	–	–
	Medium	40.4	42.9	–	40
	Severe	40.4	7.1	50	60
	Extreme	15.4	–	50	–
S-C (0–48)	Mild	17.3	50	–	–
	Medium	40.4	35.7	–	50
	Severe	34.6	14.3	50	30
	Extreme	7.7	–	50	20
Daily living (0–20)	Mild	7.7	42.9	–	20
	Medium	30.8	42.9	16.7	60
	Severe	44.02	14.3	33.3	10
	Extreme	17.3	–	50	10
Total (0–104)	Mild	9.6	71,4	–	–
	Medium	82.7	28.6	66.7	100
	Severe	7.7	–	33.3	–
	Extreme	–	–	–	–

Daily living: the Hanefeld variants were placed in 33 % of the serious and in 50 % among the worst forms; the Z-Rett presented better than the other with significant statistical difference (χ^2^ test = 25.04; *p* < 0.01).

Total: there were significant differences in the PBZ scores between different forms: 71.4 % of Z-Rett girls presented a mild score while the majority of the girls presented a medium score (χ^2^ test = 39.35; *p* < 0.001).

### Cardiorespiratory evaluation

#### Respiratory abnormalities and cardiorespiratory phenotypes

The originality of our study consists in the evaluation of the autonomic system through the use of Neuroscope which is non-invasive procedure, and provides important information on respiratory and heart parameters, and simultaneous video EEG registration electroencephalographic [15] RTT individuals who carried out the assessment with the neuroscope were 135 /151.

Thirteen abnormal, awake breathing rhythms have been identified in the RTT syndrome- see Methods section.

On the basis of the neurovegetative different features (vagal tone, heart rate, etc..), of the data of the hemogasanalysis and of the rate of the breath abnormalities, we define three different cardiorespiratory phenotypes: Apneustic, Feeble, Forceful [16]. Apneustic breathing is characterized by the prevalence of fragmented breath, in which isolated breath hold or regular and prolonged respiration dominate. Feeble breathing is characterized by the prevalence of a superficial and fast respiration during most of the time. Peculiarity of the Forceful breathing is the prevalence of hyperpnea, tachypnea and deep respiration. The recognition of the different cardiorespiratory phenotypes is important since each of them determines different metabolic consequences and determine different treatments [17]. We performed additional statistical analysis taking into account the age of the subjects in the sample. We considered three age groups: 0–5 years, 6–14 years old, and over 14. We found that the cardiorespiratory phenotypes apneustico and forced were stable over time, while the phenotype feeble increased significantly (chi2 = 23:32 *p* = 0.001). This trend is in agreement with data in literature [15] which showed that individual respiratory abnormalities (hyperpnea, apneas and breath hold) are more important in younger girls and decline over time, while shallow more represented in older females (third group).

Below we report the percentage of abnormal breathing for each clinical form (Table 13).

**Table 13:** Abnormal breathing and clinical forms

% Breath abnomal	Classical (*n* = 86)	Z-RTT (*n* = 19)	Hanefeld (*n* = 12)	Congenital (*n* = 1)	ARTT-NOS (*n* = 17)
Mean (SD)	50.8 (21.4)	41.7 (17.7)	57.1 (23.1)	37.4	42.5 (24.4)
Range	0–100	3–71.5	17.2–84.4	–	0–77

There is no significant correlation between percentages of abnormal breathing and clinical forms. Considered the importance of the breathing phenotype in RTT, we investigated how different parameters correlate to the breathing phenotype starting with BMI. For each patient, we correlated the breathing phenotype determined with clinical assessment with the BMI (Table 14).

**Table 14:** BMI centiles and cardiorespiratory phenotype

BMI	Apneustic (*n* = 14)	Feeble (*n* = 50)	Forceful (*n* = 63)
<3^rd^ centile	30 %	34 %	42 %
3rd centile–40^th^ centile	35 %	22 %	14 %
>40^th^ centile	35 %	44 %	23 %

The comparison among cardiorespiratory phenotypes and BMI was not statistically significant (*χ*^2^ test = 2.13; *p* = 0.71), however in the majority of cases (42 %) the forced phenotype presented a pathological BMI (<3^rd^ centile).

#### Cardiorespiratory phenotype and clinical forms

We considered the different cardiorespiratory phenotype in the different RTT clinical forms, and we noticed that all the respiratory abnormalities are represented in all the RTT groups but in Hanefeld variant, where we found mainly hyperpnea, tachypnea and breath-hold, while the Feeble Phenotype was lacking.

The distribution of the different cardiorespiratory phenotypes in the sample (*n* = 134) was the following: apneustic *n* = 14 (10.4 %), feeble *n* = 52 (39.5 %) and forceful *n* = 66, (48.5 %). In three cases no pathological abnormalities were present and in one case the results of the analysis were not evaluable.

Seventeen individuals could not be assessed for technical issues (Table 15).

**Table 15:** Cardio-respiratory phenotypes across clinical forms

Phenotype	Classical (*n* = 86)	Z-RTT (*n* = 19)	Hanefeld RTT (*n* = 10)	Congenital (*n* = 1)	ARTT-NOS (*n* = 18)
Feeble	37 %	53 %	–	–	50 %
Forceful	49 %	42 %	80 %	100 %	39 %
Apneustic	13 %	5 %	20 %	–	–
Normal	1 %	–	–	–	11 %

In only one classical RTT and in 2 ARTT-NOS cases no respiratory abnormalities were recorded. The following tables refer only to cases with a complete autonomic evaluation. There were no cases of Feeble phenotype in females with Hanefeld presentation.

##### Single parameters in cardio-respiratory phenotype

We looked at specific abnormalities in all the groups considered: Tachipnea, Iperventilation, Breath-hold, abnormal breathing, Valsalva Maneuver, cardiac parameters (HR and CVT) and transcutaneous parameters (PO2 e PCO2) (Table 16).

**Table 16:** Valsalva manoeuvre and clinical form

Valsalva	Classical (*n* = 86)	Z-RTT (*n* = 19)	Hanefeld (*n* = 12)	Congenital (*n* = 1)	ARTT-NOS (*n* = 17)
Mean (SD)	5.5 (9.8)	0.6 (0.7)	1.3 (1.9)	0.2 (−)	3 (7.8)
Range	0–53.2	0–2.1	0–6.8	–	0–32.8

Valsalva maneuver was independent from the cardiorespiratory phenotype. In the unique case of congenital RTT, no Valsalva’s was recorded. Valsalva’s was more common in the classical form and even so predominant to hide the cardiorespiratory phenotype [16].

##### Cardiac activity

We also looked at the cardiac activity, and the vagal tone (Tables 17, 18, 19 and 20).

**Table 17:** Heart rate (HR) by fenotype

HR	Apneustic (*n* = 13)	Feeble (*n* = 50)	Forceful (*n* = 61)	Normal (*n* = 1)
Mean (SD)	98.9 (15.8)	91.3 (15.7)	101.2 (17.4)	90
Range	74–137.4	55–130	54–148	–

**Table 18:** Heart rate and clinical form

HR	Classical (*n* = 80)	Z-RTT (*n* = 18)	Hanefeld (*n* = 12)	Congenital (*n* = 1)	ARTT-NOS (*n* = 16)
Mean (SD)	95.6 (16)	94.2 (14.4)	105.2 (24.6)	148 (−)	97.6 (13.6)
Range	55–130	65–115	54–137,4	–	78–126

**Table 19:** Vagal tone (CVT) and clinical form

CVT	Classical (*n* = 83)	Z-RTT (*n* = 19)	Hanefeld (*n* = 12)	Congenital (*n* = 1)	ARTT-NOS (*n* = 17)
Mean (SD)	5.6 (3.5)	5.9 (2.4)	6.1 (7.7)	0.7 (−)	6.4 (3.4)
Range	1.2–20	3–13.1	0.9–28.9	–	1.9–15

**Table 20:** Vagal tone and phenotype

CVT	Apneustic (*n* = 13)	Feeble (*n* = 51)	Forceful (*n* = 64)	Normal (*n* = 2)
Mean (SD)	5.5 (2.1)	5.6 (2.4)	9.2 (8.1)	9,2 (8,1)
Range	0.9–9.4	1.2–116	3.5–15	3.5–15

There was a significant difference between forceful and feeble fenotype (F test = 3.08; *P* < 0.005).

The average heart rate frequency recorded in our protocol was 96.7 bpm. We did not find significant differences.

The average value at rest for vagal tone (CTV) was 5.88.

We found differences in the CVT among different groups, with a significant abnormality in feeble and apneustic versus forced (F = 5.80; *P* <0.005 Anova with Bonferroni). Individuals with forced phenotype showed values closer to standard [15].

Individuals with forced phenotype had the lowest values of both PO2 (means 90.2; SD 13.3), and PCO2 (means 33.5; SD 12.8); while the weak phenotype and the apneustic phenotype had on average the highest levels of PCO2 and sometimes episodes of hypercapnia.

### Genetics

1. General consideration
  In the majority of cases (132 cases, 87,4 % of the subjects of the sample), the genetic mutation causative of the clinical picture was identified; in 19 cases (12,6 %) all the molecular analysis (of the *MECP2* gene and, in case of evocative phenotype, of the CDKL5 gene) resulted negative.
  Among the 132 mutated cases, a *MECP2* gene mutation was identified in 118 (89.4 %); 116 are females and 2 are males. In this group we found: 42 cases with missense mutations (36 %), 31 cases with nonsense mutations (26 %), 28 with frameshift mutation (24 %) and 17 with large deletions (14 %). In particular the mutations identified in the two males were one missense mutation (p. Arg306His, in a mosaic state) and one frameshift (p. G269fs).
  Furthermore, there were 12 cases with a mutation in the *CDKL5* gene (9.0 %), 1 case with a mutation in *FOXG1* (0.75 %) and 1 case with a mutation in *MEF2C* (0.75 %).
  In this study we found a significant correlation between C-terminal and the Z-RETT variant [21].
2. Different clinical RTT forms and genes involved (Table 21)

**Table 21:** Clinical forms and genes

	Classical	Atypical				Total
		Hanefeld	Congenital	Z-RTT	ARTT-NOS	
RTT male MECP2 +	–	–	–	–	2/20 (10 %)	2
RTT females MECP2 +	94/98 (96 %)	–	–	18/19 (95 %)	4/20 (20 %)	116
RTT females MECP2-	4/98 (4 %)	–	–	1/19 (5 %)	13/20 (65 %)	18
CDKL5 +	–	12	–	–	–	12
CDKL5-e MECP2-		1				1
FOXG1 +	–	–	1	–	–	1
MEF2C +	–	–	–	–	1/20 (5 %)	1
TOTAL	98 (100 %)	13 (100 %)	1	19 (100 %)	20 (100 %)	151
  Among the classical RTT and the Z-RTT, a *MECP2* mutation was found in almost the majority (respectively 96 % for classical and 95 % for Z-RTT) of the cases. In the last 4 cases of classical RTT and 1 case of Z-RTT no *MECP2* or *CDKL5* mutations were found.
  We did not find *MECP2* mutations in cases with either congenital or Hanefeld variant. The only congenital RTT had a *FOXG1* mutation and all the Hanefeld RTT variant cases had *CDKL5* mutation, confirming the correlation between *CDKL5* mutations and Hanefeld variant.
  In the group of the ARTT-NOS, a *MECP2* mutation was identified in only 6/20 (30 %) of the cases, suggesting that this group is probably heterogeneous in term of etiology.
  In individuals of the ARTT-NOS group, the *MECP2* positive girls had a very early onset of the neurological symptoms without evidence of regression. The mutations identified in this group were: p. Arg106Trp, p. Pro251Arg, p. Arg255Ter. p. G269fs.
3. MECP2 positive cases and RTT clinical forms
  In the whole sample, almost all the mutations types were represented (Table 21): the most frequent mutations group were the missense mutations and the less frequent were large deletions.
  The frameshift mutations, are mostly (68 %) in the C terminal region (Table 22).

**Table 22:** MECP2 mutations and clinical forms

Mutations	Classical	Atypical		Tot	%
		Z-RTT	ARTT-NOS		
Substitutions					
Non sense	29	1	1	31	26 %
Early truncating	22	–	1	23	
Late truncating	7	1	–	8	
Missense	29	9	4	42	36 %
Large Deletions	17	–	–	17	14 %
Frameshift	19	8	1	28	24
*C terminal deletions*	*12*	*7*	–	*19*	
	94	18	6	118	100 %
  We did not observe any significant statistical correlation among the different MECP2 mutations and the different RTT clinical forms, with the exception of the predominance of C-terminal deletions in Z-RTT variants.
  In our sample, the eight recurrent mutations (p. Arg106Trp, p. Arg133Cys, p. Thr158Met; p. Arg168Ter; p. Arg255Ter; p. Arg270Ter, p. Arg294Ter, p. Arg306Cys) representing in literature about the 80 % of the *MECP2* mutations [20], account for about 47 % of the total RTT MECP2 mutated cases. Among the differents RTT clinical forms, in our sample the majority (60 %) of the p. Arg106Trp cases had a clinical picture fulfilling the criteria for Z-RTT.
  About the other types of mutations, we found a relative higher frequency of large deletions (14 % of cases, all in classical RTT) and C terminal deletions (16 % of cases).
  c) MECP2 mutations and ISS
  Statistical analysis showed that ISS score is lower in the group of the C terminal deletions RTT girls if compared to the other mutations groups [21] (Table 23).

**Table 23:** ISS total score and MECP2 mutations

ISS total	Early truncating (*n* = 22)	Late truncating (*n* = 8)	Large deletion (*n* = 18)	Frameshift (*n* = 9)	Missense (*n* = 39)	C ter del (*n* = 18)
Mean (SD)	21.3 (6.2)	16.7 (4.7)	19.2 (5.9)	20.3 (7)	18.4 (5.7)	14.2 (6)
  No other significant correlations was found among other type of mutations (early truncating mutations, late truncating mutations, deletions) and ISS clinical score.
  We investigated correlation between different *MECP2* mutations and the ISS scores, and we found a significant correlation between C-Terminal mutations and ISS domains development and autonomic functions:
  Development: 77 % of the mutations terminals C had a mild severity (ISS) for domain DEV (development and growth) while all other mutations were mild only in 33 % of cases (test *χ*2 = 7.63; *p* < 0.05).
  Autonomic system: 72 % of cases C term were mild compared to 33 % of other (*χ*2 test = 9.78; *p* < 0.01).
  Total: 78 % of cases the term C had a mild form versus 24 % of other forms (*χ*2 test = 3.20; *p* < 0.001).
  d) MECP2 mutations and PBZ
  Like for ISS, we found that showed that the group of the C terminal deletions RTT girls has lower PBZ score if compared to the other mutations groups. This is true for the total score and the motor abilities (Table 24).

**Table 24:** PBZ total score and MECP2 mutations

PBZ total	Early truncating (*n* = 9)	Late truncating (*n* = 5)	Deletions (*n* = 13)	Frameshift (*n* = 3)	Missense (*n* = 24)	C terminal del (*n* = 14)
Mean (SD)	34.4 (13.5)	33.4 (9.3)	38.2 (8.8)	42.7 (11.6)	32.6 (9.9)	23.4 (12)
  MOTOR abilities: 57 % of the C terminal del. cases had a milder presentation (19 %) compared to other groups (*χ*^2^ test = 28.52, *p* < 0.05).
  DAILY LIVING: 86 % of C terminal del. cases had mild or medium presentation.
  TOTAL score PBZ: 50 % of C terminal del. cases had a presentation mild–medium versus 13 % of the non C terminal del. forms (χ2test = 9.77, *p* < 0.01).
  e) MECP2 C terminal deletion phenotype
  Overall we had 19/118 cases with a C terminal deletion. Twelve of them (63 %) had a RTT classical form and seven (37 %) had a Z-RTT form [6, 22, 23].
  The age-range is from 2 to 20 years. Growth parameters were in the normal range in almost all the girls (except one case for which data are not available). OFC was in the normal range in about 10/19 cases, in 6/19 between the 3rd and 40 % centile and only in 2 cases females showed clear microencephaly.
  Total ISS score evaluation showed for C terminal deletion cases the majority (13/19) had a mild score, 4/19 case had a moderate score and in 1/19 case a severe score. Epilepsy was absent in 8/19 cases, present but drug controlled in 10/19 cases and only in 1/19 case the epilepsy was drug resistant. Scoliosis was absent in the 8/19 cases and present but of a mild degree in 7 cases. No one RTT girls needed a surgical intervention for scoliosis. Only 4/19 were not able to walk, while 9/19 walked with a support and 6/19 were able to walk alone.
  Tremors or tremors like were present in half of this sample. In almost all cases, hands stereotypies were present but non dominant. In 6 of them (range 7–20 years of age), all with Z-RTT, the language was composed by >20 words. The cardiorespiratory phenotype was forced in 9 cases, feeble in 8 cases, and apneustic in 1 case; 1 case was not analysed.
4. CDKL5 positive cases
  Overall we had 13/151 cases with Hanefeld variant who all but one presented *CDKL5* gene mutations. All the cases belonging to this group can be defined as Hanefeld variant RTT. They had a age-range of 1–12 years with a mean age of about 6 years.
  Total ISS total score evaluation showed that Hanefeld variants had higher severity than all the other RTT forms. According to the PBZ evaluation scales, the scores for the *CDKL5* cases were higher (severe) than the *MECP2* one (F test = 3.18; *p* < 0.005) and this is mainly regarding the sensorial and communicating abilities (F test = 5.88; *p* < 0.005) and Dayly living.
  Considering the ISS different subscales, the Muscolo-skeletal area was the most affected if compared to the others. In the growth domain, both height and weight were in the normal range in the majority of cases (with the height at the upper centile). OFC was below the 3^rd^ centile in 8 % of cases, a portion comparable with what have been observed in Z-RTT. BMI was better than what observed in all the RTT form (with the exception of the Z-RTT which is less compromised). Scoliosis was absent in the majority of cases (75 %).
  Walking was difficulty in about 84 % of cases, of which half of them was not able to walk without support. Hand stereotypies, mainly of mild degree, were present in all the samples. Hand dyspraxia was more severe in the group of the Hanefeld variant (together with classical RTT, ART-NOS) if compared to the Z-RTT.
  Epilepsy was present in all the cases (100 %) and there was a higher percentage of drug resistant cases compared to all the other RTT subgroups. The median age of onset was two, 2 months with a range from 1 to 7.
  Among gastrointestinal problems, dysphagia was present in 61 % of cases (with mild degree in the half of cases) and constipation in about the 76 %. Only a small subgroup presented a severe sleep disorder while the majority had only a mild impairment.
  The cardiorespiratory phenotype was forced in the majority of cases. No Feeble phenotype was found [9].

## Discussion

The present study was conducted on a population of individuals with Rett syndrome who regularly attended the Tuscany Rett Center. The majority of the cohort is represented by girls living in the same region, but we account a considerable number of girls, and even males with Rett syndrome, coming from various parts of Italy. Hence this is the first study conducted in Italy on a large, unbiased, group of individuals affected by this condition.

The cohort has classic cases as well as variants [4]. We account for a considerable percentage of atypical forms (35 %), greater than data reported in the literature, included between 15 and 30 % [24–26]. MECP2 mutations were present in almost all classical and Z-RTT cases, a notable link between these cases where clinical features slowly change from one end to another. The ARTT-NOS show different kind of genetic abnormalities in only 30 % of the cases.

All individuals fulfilled the diagnostic criteria for Rett Syndrome in the classical form or variants: all individuals had a delay in development, different degree of regression and an autistic phase. In 20 % of cases autistic traits were marked even years after diagnosis. The sample consisted of 151 individuals (all females except 2 cases), of which 132 subjects with genetic mutations: 118 with *MECP2* mutations; 12 with *CDKL5* mutations 2 with *FOXG1* and *MEF2C* mutations. For 19 individuals with clinical diagnosis (12.5 %) no genetic mutation was identified.

Considering the clinical symptoms described in classic cases and in those with the preserved speech variant (Z-RTT) and the corresponding *MECP2* mutations, we observed a parallel, progressive sequence in the severity of symptoms, concerning the terminal C deletion in several cases of Z-RTT variant and in a more limited number of classic cases only. Somatic features varied from a minor signs in classic cases to normal physical appearance in Z-RTT; scoliosis followed the same pattern and the same applies to the ability to walk and the use of hands. Hand stereotypic activities (‘hand washing’) were intense and present all day in the waking state in classic individuals and became occasional in Z-RTT; the same applies to epilepsy as well as to cardiac and breathing abnormalities, more frequent and intense in severe cases and less pronounced in those with some speaking ability. In contrast, we observed that intensity of general anxiety is inversely correlated with clinical severity [27]. In all these cases a constant regression followed an initial period (several months) when the toddler did not show clear signs of abnormality and was usually considered to be normal by parents although abnormalities of General Movements could be detected by professionals [28, 29]. In the present study Z-RTT was more frequently observed (12.6 %) than in previous studies as, for example, only 3 out of 168 individuals in Spain [30].

It has recently reported [31] that most cases of *FOXG1* mutation have structural brain abnormalities including corpus callosum malformation, present also in the only congenital case of the present study. The study suggests that this is a distinct clinical entity with different features and the hypothesis is further supported by lack of scoliosis and an entirely different clinical course. In these cases symptoms appear soon after birth and, in contrast with classical and Z-RTT, don’t have a regression. Some authors associate these cases to the dyskinetic encephalopathy of infancy [32]. A similar suggestion has been made for the early seizure variant (Hanelfeld) with a different clinical appearance where females present minore frequenza of scoliosis and a persistent gaze avoidance; in addition, in most girls regression occurs in the context of severe seizures. This is different from typical Rett, rare *MECP2* mutations and in most case a different mutation *CDKL5*, data which correspond to a large extent to what has been observed in the present study [33, 34].

In our sample we considered both the age of the evaluation and the age of diagnosis: this data are a measure of the time required to make the diagnosis of Rett syndrome, which is different for each clinical form [33], and not necessarily directly correlated to the age of onset of symptoms, but is linked to the complexity of the diagnosis. Important symptoms such as developmental delay, absence and / or loss of language, presence of stereotypies and eating disorders favour the early diagnosis; absence of stereotypies, presence of language, autistic traits and mild motor disorders, lead specialists to incorporate these cases into broader diagnostic categories such as mental retardation or autism and contribute to delay the correct diagnosis [26], similarly to what observed in atypical presentations [35]. In our study, the frequency of labeled autistic traits was present in the 20 % of individuals.

### Autonomic nervous system (ANS)

A major contribution of this study is connected to the attention paid to disorders of the autonomic nervous system, which are present in all clinical forms, although with some variability.

Imbalances of ANS, essentially characterized by a vagal tone comparable to that of a newborn and therefore particularly immature [17] implies a constant imbalance of sympathetic parasympathetic activity in favour of the latter and determines the following symptoms: impaired swallowing, abnormalities in the gastro-esophageal tract and urinary bladder, mood disorders, sleep disorders, peripheral circulation in the limbs and abnormalities of breathing frequency.

All clinical forms presented massive respiratory dysfunctions which, although with great variability across single cases, affected 50 % of the whole registration time for the classic forms and 40 % of the Z-Rett and RTT-NOS individuals. The other two variants had higher values: 57.1 % in Hanefeld’ s and 37.4 % in the congenital forms; these differences were not statistically significant.

We found cardiorespiratory abnormalities in all our subjects with classic RTT, with the exception of one single case, and two females in the ART-NOS group. The Valsalva maneuver is present in the classical forms and represents 5.5 % of all respiratory abnormalities; in the Z-Rett the maneuver is almost always absent (0.5 %). There were no differences for HR, CVT, and blood gases that depend more on the cardiorespiratory phenotype than on the clinical form. For the CVT analysis, the study showed high values in individuals with forceful breathing (never described in literature observation). In particular, the values of vagal tone (CVT) in the individuals with forceful were closer to the control values than the CVT of the other individuals. Heart rate, on the contrary, did not show significant differences across different phenotypes [17].

The comparison of cardiorespiratory phenotypes showed higher levels of PCO2 in feeble and in apneustic phenotype than in forced phenotype. This observation has particularly important neurophysiological implications, both in the event of general anesthesia and in cases where, in the worsening of the respiratory disorder, the organism generates a pathological change of the CO2 (either an increase, or a reduction: hypo- and hypercapnia) [18].

Gastrointestinal phenotype was compromised, and severe feeding disorders required in some cases the use of some devices, like percutaneous endoscopic gastrostomy (PEG), useful for long period of time, or the nasogastric tube, for short period of time [36]. In our study, the use of PEG was limited to 3 cases.

To further define the differences in the symptoms related to ANS between the clinical forms, we considered sleep. The prevalence of sleep disorders is particularly high in Rett population, in order of 80 % [37]; daytime napping, night-time laughter, teeth grinding, screaming and night awakenings due to the appearance of seizures were the most frequently reported problems. The major problems were related to difficulty falling asleep or night-time/early morning awakenings. In our cases, the measurement time, representing 38 % was more frequently associated with Hanefeld through against the classic forms and Z-Rett.

Comparison between scoring systems ISS and PBZ: the ISS highlights the reduced impairment of Z-Rett compared to all other forms with statistically significant differences in the overall assessment, as in growth and development area, in the muscle skeletal, in the area of movement and in the cortex. However, it does not show significant differences across all other dependent forms of the Autonomic Nervous System, in contrast to what reported in literature (see Table 12).

The results obtained with the two scoring systems ISS and PBZ are comparable, and PBZ evaluation allowed an evaluation of the daily living. The PBZ is a scale of evaluation designed to test severity in Classical Rett Syndrome and its variants. The scale is susceptible to recording even minimal changes in clinical symptoms over the course of therapy, rehabilitation programmes and environmental enrichment, but also to indicating the severity of different clinical and genetic groups [14]. The evaluation with PBZ scoring system showed a worse clinical presentation for Hanefeld variant; daily living evaluation greatly contributed to the evaluation of the phenotype. Even with the PBZ system the Z-Rett was the mildest form in our cohort for all the areas considered. All the reported differences were significant in each area.

In general, the PBZ scale allows a more sophisticated classification. In fact ISS system allows only two values for defining the cognitive level and language: severe and extreme, but it does not accept that there is no sign of Rett, even though there are some females with Z-Rett, with a good speech and motor performance.

Both PBZ and ISS scales showed a better score for the auxological parameters in the Z-Rett and Hanefeld girls, for all the considered parameters (height, weight, and OFC). The BMI values were significantly different in favour of any other forms, Hanefeld included. The BMI did not correlate to the clinical form, but was associated with cardio-respiratory phenotype: this is consistent with an association between low BMI and forced phenotype because of the energy consumption in this phenotype. The data are consistent with those reported in the literature: mean weight, height, and BMI z scores in subjects with Rett syndrome were below that of their age group and decreased at steadily with age [38].

Cortical activity is clearly related to the onset and severity of epilepsy. Epilepsy was present in 64.2 % of the cases, a value close to the 68.1 % reported from the Rett network database. Drug resistant cases were less frequent: 17.2 % compared to 32.2 % of the above quoted study [39] possibly related to a comparatively greater number of Z-RTT girls where drug resistant epilepsy in unusual. Also, the onset of epilepsy occurs earlier in Hanefeld. In all the females with *CDKL5* mutation presented major sleep disruptions compared to other forms: RTT versus other forms: *χ*^2^ test = 83.399; *p* = 0.0001; ARTT-NOS vs Hanefeld vs classical vs congenita: *χ*^2^ test = 4.82; *p* = 0.18 not significant. It is possible that in our cohort, the higher number of Z-Rett girls justify some of the differences observed between our and other classifications- for example, the resistant epilepsy described by Krajnc [40].

Finally, all clinical forms considered were indistinguishable from each other as regards the ANS involvement, but overall the scales used confirmed that the Z-RTT had milder phenotype if compared to all the other forms, and have the best quality of life as assessed with the PBZ (*χ*^2^ test = 25.04; *p* < 0.01).

Genetic analysis showed that there is a correlation between gene involved and different clinical forms but there is a weak correlation between the severity of the phenotype and type of mutation, except for C-Terminal deletion that were statistically associated with clinical forms with the lowest scores of severity scales (ISS and PBZ) in accordance with what is reported in the literature [41, 42].

## Conclusions

The present study confirms the variability of Rett clinical phenotype and adds new autonomic and anthropometric variables to clinical data with better results in the Z-RTT girls. Autonomic abnormalities were common in the classical form and present also in a number of cases of the preserved speech variant. In this respect it must be noticed that abnormalities of this kind are common also in other neurodevelopmental disorders and have been described in Autistic Spectrum Disorders [11].

In our cohort we found a broad variety of RTT clinical forms. In the great majority of individuals with classical form and preserved speech variant there was a mutation in the *MECP2* gene with a correlation between clinical evaluation and genetic analysis. Almost all cases of Hanefeld variant had a *CDKL5* mutation. There was only one case of the congenital form.

An analysis of the *MECP2* mutations, showed that C-terminal mutations were associated to milder clinical forms and lower scores in severity scales (ISS and PBZ), confirming what has been observed in other studies [41, 42].

However, considered the spectrum of all *MECP2* mutations, females with the Z-Rett variant present a milder phenotype and severity seems to be related to the early development of the disorder, suggesting that the natural history of the disease is dependent to the stage of regression: absent or reduced.

The frequency of epilepsy was not different across different clinical forms, although we did not find any patient with drug-resistant epilepsy in the Z-Rett females. Other authors report a decreased frequency of epilepsy in Z-Rett, but in our cohort the number of females with Z-Rett was higher than in other studies and this can influence the final outcome [39, 40].

We did not find significant correlation between clinical forms and cardio-respiratory phenotype: however, the forced breathing phenotype was associated to a reduced BMI, suggesting that the high energetic cost of this abnormal breathing leads to a chronic hypoxia.

The breathing abnormalities are not typical of Rett syndrome, however the breathing patterns are an essential element of the diagnosis, for example, the evaluation of the ANS allows the distinction between non-epileptic paroxysm and epilepsy, contributes to the diagnosis in MECP2 negative forms, allows to design a personalized therapy [17], and eventually, taking into account all the limitations, a better prognosis.

Similarly, we did not find a significant correlation between clinical form and cardio-respiratory phenotype, however the forceful breathing phenotype was associated to high CVT values and to a reduced BMI suggesting that the high energetic cost of this abnormal breathing leads to a chronic hypoxia. The non-invasive assessment of brainstem function in Rett may contribute to life-long clinical management and might be as important as the genotype in understanding the pathology and in prognostication.

## Abbreviations

ARTT-NOS: atypical RTT-not otherwise specified
BMI: body mass index
Brain: autonomic function
CDKL5: cyclin dependent kinase-like 5
CORT: cortical
CVT: cardiac vagal tone
DEV: growth and development
FOXG1: forkhead box g1
ISS: International Scoring System
MECP2: methyl CpG binding protein 2
MOV: movement
M-Skel: musculoskeletal appearance
OFC: occipital frontal circumferenc
PBZ: Pini Bonuccelli Zappella scale
PEG: percutaneous endoscopic gastrostomy
RTT: rett syndrome
S-C: sensorial and communicative abilities
Z-RTT: preserved speech variant or “Zappella variant”
